# DNA Methylation Panels for the Differentiation of Lung and Gastric Adenocarcinomas from Other Common Primary Adenocarcinomas

**DOI:** 10.3390/cancers16234000

**Published:** 2024-11-29

**Authors:** Tina Draškovič, Lara Omahen, Maja Jerše, Nina Zidar, Nina Hauptman

**Affiliations:** Institute of Pathology, Faculty of Medicine, University of Ljubljana, Korytkova 2, 1000 Ljubljana, Slovenia; tina.draskovic@mf.uni-lj.si (T.D.); lo9571@student.uni-lj.si (L.O.); maja.jerse@mf.uni-lj.si (M.J.); nina.zidar@mf.uni-lj.si (N.Z.)

**Keywords:** methylation biomarkers, gastric adenocarcinoma, lung adenocarcinoma, hepatocellular carcinoma, cholangiocarcinoma, colorectal carcinoma, pancreatic adenocarcinoma, adenocarcinoma differentiation, DNA methylation

## Abstract

**Background/Objectives**: Gastric and lung adenocarcinomas are among the most common adenocarcinomas worldwide. Our research aimed to validate methylation biomarkers that differentiate gastric and lung adenocarcinomas from hepatocellular carcinoma, cholangiocarcinoma, colorectal carcinoma, pancreatic adenocarcinoma and paired healthy tissues. **Methods**: The study analyzed 178 formalin-fixed, paraffin-embedded tissue samples, including 14 gastric adenocarcinomas, 15 lung adenocarcinomas, 15 hepatocellular carcinomas, 15 cholangiocarcinomas, 15 colorectal carcinomas, 15 pancreatic adenocarcinomas and their paired healthy tissues. Methylation status was determined experimentally by methylation-sensitive high resolution melting. The diagnostic panels were validated on bioinformatics datasets from The Cancer Genome Atlas and Gene Expression Omnibus, comprising 1981 and 773 samples, respectively. Sensitivity, specificity, diagnostic accuracy and predictive values for each cancer type were calculated for the experimental, Gene Expression Omnibus and The Cancer Genome Atlas datasets. **Results**: The gastric cancer-specific panel showed a sensitivity of 78.6–83.9%, a specificity of 86.6–94.6% and a diagnostic accuracy of 89.9–96.1% to differentiate between all tumors, and a sensitivity of 78.6–83.9%, a specificity of 89.2–96.4% and a diagnostic accuracy of 88–96.1% to differentiate between all tumors and healthy tissues. The lung adenocarcinoma-specific panel showed a sensitivity of 61.1–93.3%, a specificity of 70.3–90.8% and a diagnostic accuracy of 74.2–90.6% to differentiate between all tumors, and a sensitivity of 61.1–93.3%, a specificity of 77.9–93.4% and a diagnostic accuracy of 79.2% to 93.1% to differentiate between all tumors and healthy tissues. **Conclusions**: This study demonstrates the potential of using diagnostic methylation panels to differentiate gastric and lung adenocarcinomas from other common adenocarcinomas and paired healthy tissues.

## Simple Summary

This study aimed to validate diagnostic panels for gastric and lung adenocarcinomas by differentiating them from hepatocellular carcinoma, cholangiocarcinoma, colorectal carcinoma, pancreatic adenocarcinoma and paired healthy tissues. A total of 178 formalin-fixed, paraffin-embedded tissue samples were analyzed using methylation-sensitive high resolution melting. The performance of the panels was further validated on 2754 samples from publicly available datasets. The gastric adenocarcinoma-specific panel showed a sensitivity of 78.6% to 83.9%, a specificity of 89.2% to 96.4% and a diagnostic accuracy of 88% to 96.1%. The lung adenocarcinoma-specific panel showed a sensitivity of 61.1% to 93.3%, a specificity of 77.9% to 93.4% and a diagnostic accuracy of 79.2% to 93.1%. These results emphasize the potential of using methylation panels as diagnostic tools to differentiate gastric and lung adenocarcinomas from other adenocarcinomas and healthy tissues.

## 1. Introduction

Adenocarcinomas are the most frequent malignant tumor found throughout the body. They can arise from a variety of tissues and organs, including the stomach, lungs, colon, pancreas and liver. They range from slow-growing tumors to aggressive malignancies, depending on the organ and other characteristics. Several environmental, as well as lifestyle risk factors, are associated with the development of adenocarcinomas [1].

Gastric adenocarcinoma (GAC) is one of the most aggressive malignant diseases worldwide and is responsible for 3 to 10% of cancer-related deaths. GAC is often diagnosed at an advanced stage with lymph node metastases present [2,3].

Lung adenocarcinoma (LUAD) is the most common form of primary lung cancer. Although smoking and tobacco consumption remain the main risk factors for lung cancer, this subtype often occurs in non-smokers and women. It is the second most commonly diagnosed tumor in men and women worldwide and is the leading cause of cancer-related deaths. Similar to GAC, it is often diagnosed late, when the disease has already progressed and metastasized. As a result, the five-year mortality rate remains high, ranging from 51% to 99% depending on the stage [4,5].

Advances in technology have enabled the discovery and accurate determination of genetic and epigenetic biomarkers, including DNA methylation. DNA methylation involves the addition of a methyl group to the cytosine base in DNA, which often affects gene expression without altering the DNA sequence. Differentially methylated CpGs and regions are increasingly utilized to distinguish between different types of cancer, including adenocarcinomas. Alongside their potential use in diagnostics, DNA methylation biomarkers may enable early detection of cancer and provide insights into tumor behavior, progression, prognosis, treatment selection and monitoring response to therapies [6,7].

This study includes the validation of bioinformatically identified methylation biomarkers that are LUAD-specific and GAC-specific. The bioinformatics research utilized publicly available methylation data from the Illumina Infinium HumanMethylation450 BeadChip (HM450) and the lllumina MethylationEPIC BeadChip (EPIC) for several cancer types and included more than 3600 samples. It included two approaches: a non-clustered approach and a clustered one in which unsupervised hierarchical clustering was performed. After performing differentially methylated region analysis, the study focused on identifying diagnostic DNA methylation biomarkers that are hypermethylated in the cancer of interest (in our case, we identified CpG sites that are hypermethylated in LUAD or GAC) and hypomethylated in other cancer types [6]. To bridge the bioinformatics and experimental work, probes from the HM450 and EPIC platforms—designed to target specific DNA sequences—were replicated. In this context, a “probe” refers to a short DNA sequence that binds to a complementary DNA region in a sample, enabling the detection or quantification of methylation at specific CpG sites. These probes are an integral part of the HM450 and EPIC platforms, representing precise DNA sequences associated with methylation markers. In our experimental setting, primers were designed based on these probe sequences to allow direct comparison between the methylation data generated by the HM450/EPIC platforms and the results obtained in our experiments. By retaining the term “probe,” we emphasize its association with the HM450 and EPIC platforms, and its role in targeting specific DNA sequences. This approach effectively extends the bioinformatics findings into the laboratory setting, ensuring consistency between computational predictions and experimental validation.

**Figure 1:**
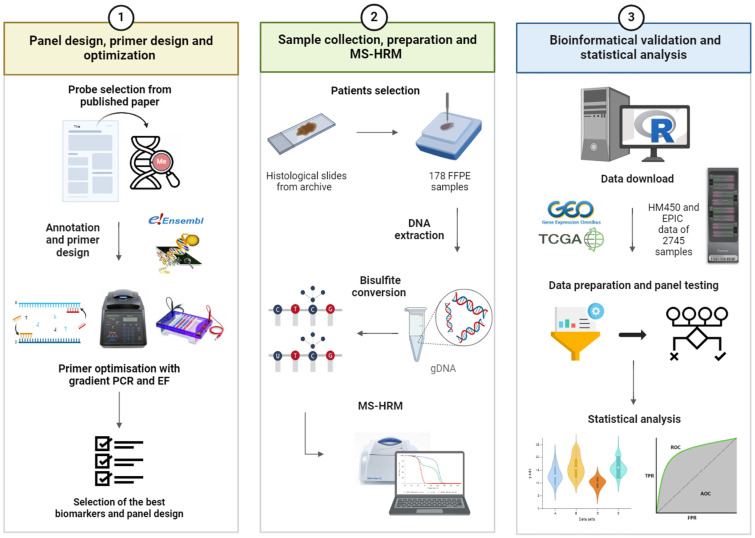
Simple study workflow. Methylation biomarkers, grouped into gastric adenocarcinoma-specific and lung adenocarcinoma-specific panels, were selected from a published bioinformatics study. After annotating the probes, primers were designed and optimized. In the bioinformatics study, numerous LUAD-specific and GAC-specific probes were identified, but only the best ones, which passed the optimization process, were combined into panels and experimentally validated on patient samples. Histologic slides and FFPE samples from selected patients were obtained from the institute’s archive. After DNA extraction, bisulfite conversion and MS-HRM were performed. After experimental validation, our results were validated using publicly available GEO and TCGA data. A statistical analysis was performed for the experimental results and the bioinformatics results. LUAD, Lung adenocarcinoma; GAC, Gastric adenocarcinoma; FFPE formalin-fixed, paraffin-embedded; MS-HRM, Methylation-Sensitive High Resolution Melt analysis; GEO, Gene Expression Omnibus; TCGA, The Cancer Genome Atlas; EF, electrophoresis; HM450, Illumina Infinium HumanMethylation450 BeadChip; EPIC, lllumina MethylationEPIC BeadChip. Created with BioRender.com.

Our research aimed to validate novel LUAD-specific and GAC-specific methylation biomarkers that successfully differentiate LUAD and GAC from cholangiocarcinoma (CCA), colorectal carcinoma (CRC), hepatocellular carcinoma (HCC), pancreatic adenocarcinoma (PDAC) and the normal adjacent tissues (NATs). A schematic flow of the study is presented in Figure 1.

## 2. Materials and Methods

### 2.1. Patient Cohort and Sample Selection

The study utilized formalin-fixed, paraffin-embedded (FFPE) tissue blocks for analysis. The study included samples from patients with various types of adenocarcinomas. These cases were selected based on histological slides of resected tumors between 2017 and 2023, which were reevaluated for the purpose of the study by two certified pathologists. The final diagnosis and the pathological features of the samples were confirmed, and the area in each sample that best represented the selected diagnosis was selected. Areas with inflammation and immune cell infiltration, as well as areas with necrosis, were avoided. In order to achieve the most accurate sampling, the selected areas of the FFPE tissue blocks were punched. All samples were retrieved from the archives of the Institute of Pathology, Faculty of Medicine, University of Ljubljana. The selected samples included 14 GACs, 15 LUADs, 15 HCCs, 15 CCAs, 15 CRCs and 15 PDACs. Paired normal adjacent tissues (NATs) were also chosen for further analyses, resulting in a total of 89 pairs of primary solid tumors (TP) and NATs. The demographic data of 89 patients, including sex, age, disease category, histologic subtype, histologic grade and TNM classification of malignant tumors, are presented in Supplementary Table S1. The dataset consists of 48 men and 41 women with an average patient age of 67.2 years. The research was conducted in accordance with the ethical standards of the Helsinki Declaration and was approved by the National Medical Ethics Committee of the Republic of Slovenia (Approval No. 0120-485/2020/5).

### 2.2. DNA Extraction and Bisulfite Conversion

Total DNA was extracted using the automated Maxwell^®^ RSC FFPE Plus DNA Kit with the Maxwell^®^ RSC Instrument (Promega Corporation, Madison, WI, USA). Isolation followed the manufacturer’s instructions, utilizing 3–6 FFPE punches from each paraffin block. The concentration of the isolated DNA was measured spectrophotometrically using a NanoDrop One Spectrophotometer (Thermo Fisher Scientific, Waltham, MA, USA) and a Qubit™ 4 Fluorometer. Bisulfite conversion of the DNA was performed using the MethylEdge^®^ Bisulfite Conversion System (Promega Corporation, Madison, WI, USA), following the manufacturer’s instructions, resulting in a final eluted concentration of 20 ng/µL.

### 2.3. MS-HRM Conditions and Analysis

For the methylation-sensitive high resolution melt (MS-HRM) experiment, primers were designed using Methyl Primer Express Software v1.0 (Thermo Fisher Scientific, Waltham, MA, USA), based on the bisulfite-converted DNA sequence, to amplify both methylated and unmethylated DNA. These primers were specifically designed to target CpG sites that best discriminate between gastric and lung adenocarcinomas from other common adenocarcinomas, as identified in our bioinformatics study [6]. To identify the optimal annealing temperature for each primer set, gradient PCR was performed on a Gradient DNA Engine Thermal Cycler (Bio-Rad, Hercules, CA, USA), and the PCR products were analyzed using 2% agarose gel electrophoresis. The annealing temperature that produced the strongest band on gel electrophoresis was selected for the MS-HRM experiment and is listed in Table 1.

Further optimization was carried out using the Rotor-Gene Q Thermocycler (Qiagen, Hilden, Germany), following the manufacturer’s recommendations. Control DNA samples with known methylation statuses were used to determine different profiles: fully methylated control DNA, 30% methylated control DNA (a mix of methylated and unmethylated control DNAs) and unmethylated control DNA (EpiTect PCR Control DNA Set, Qiagen, Hilden, Germany).

**Figure 2:**
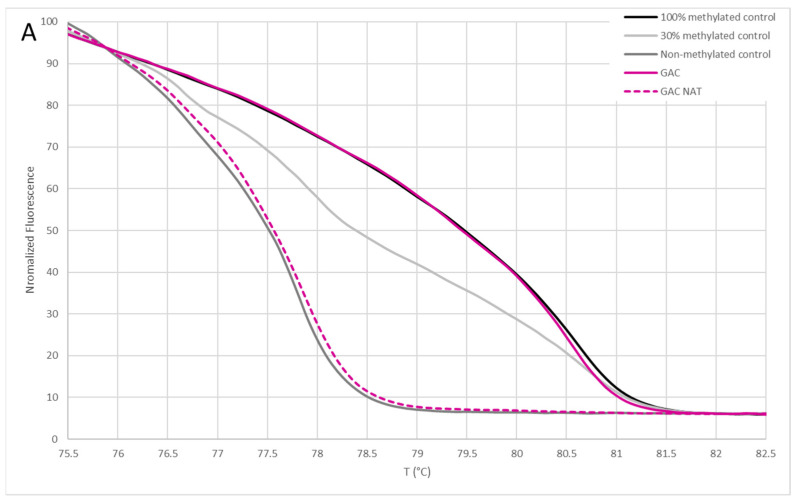

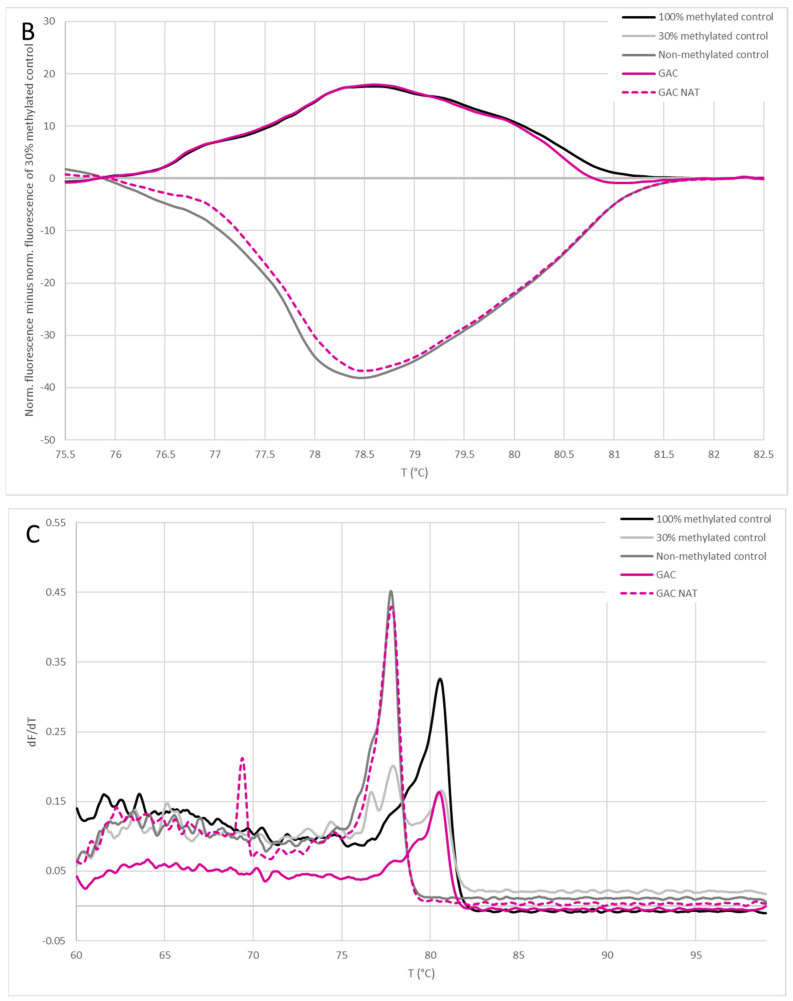
An example of MS-HRM experimental results for the cg18542829 methylation site in the *ABCB1* gene, included in the gastric adenocarcinoma panel. Results are shown for a gastric adenocarcinoma sample, a normal adjacent tissue sample and methylation controls. (**A**) Normalization plot, (**B**) Difference plot and (**C**) Melt curves. GAC, gastric adenocarcinoma; NAT, normal adjacent tissue.

The reactions included HotStarTaq Plus DNA Polymerase (Qiagen, Hilden, Germany), HotStarTaq Plus Buffer (Qiagen, Hilden, Germany), dNTPs, SYTO9 (Thermo Fisher Scientific, Waltham, MA, USA) fluorescent dye and nuclease-free water in a final volume of 10 µL. The PCR conditions were as follows: initial denaturation at 95 °C for 5 min, followed by 45 cycles of denaturation at 94 °C for 15 s, annealing at the selected temperature (Table 1) for 30 s and extension at 72 °C for 30 s. High resolution melting analysis was subsequently performed, with temperatures ramping from 60 °C to 99 °C, increasing by 0.1 °C every 2 s. Each run included bisulfite-converted DNA from the samples, methylated control DNA, 30% methylated control DNA and unmethylated control DNA. The methylation status of GAC, LUAD, HCC, CCA, PDAC, CRC and their NAT samples (unmethylated/methylated) was determined by comparing the normalized melting curves and/or difference plots of the tested samples with DNA methylation controls of known methylation status. If the normalized melting curve profile of the samples was equal to or higher than the profile of the 30% methylated DNA control, the sample was labeled as methylated, otherwise it was labeled as unmethylated (Figure 2A). The cut-off value of 30% methylation was selected based on published bioinformatics work, from which methylation biomarkers were selected, and is supported by other publications [6,8,9,10]. When the 30% methylation control is set to zero in the difference plot, samples with methylation levels above 30% appear above the control, while those with methylation levels below 30% fall below it (Figure 2B). For additional validation, melt curves were consistently analyzed (Figure 2C). In these curves, the peaks for unmethylated DNA and 100% methylated DNA controls are distinct and clearly separated. Samples labeled as unmethylated displayed a peak preceding that of the methylated samples.

### 2.4. Bioinformatics Analysis

To prove the reproducibility and validity of the MS-HRM results, we validated biomarkers and panels on Gene Expression Omnibus (GEO) and The Cancer Genome Atlas Program (TCGA) datasets. The methylation array-based beta values of the HM450 and EPIC arrays were used to represent the methylation status of the samples. The GEO datasets GSE113017 [11], GSE113019 [11], GSE75041, GSE217384 [12], GSE201241 [13], GSE49656 [14], GSE220160, GSE119526 [15], GSE149282 [16], GSE159898 [17], GSE77954 [18], GSE53051 [19], GSE63704 [20], GSE134217 [21], GSE207846 [22], GSE103186 and GSE99553 [23] were included. When available, the raw data were downloaded from GEO and processed, including quality control, normalization and calculation of the beta value with the minfi package [24]. Only samples that passed the quality controls were retained for analysis. For projects without raw data, we relied on the processed data provided by the original authors where quality control and normalization had already been conducted. These datasets were downloaded and the beta values were extracted using the GEOquery package [25]. For the TCGA dataset, the data for the cholangiocarcinoma (CHOL), colon adenocarcinoma (COAD), hepatocellular carcinoma (LIHC), LUAD, pancreatic adenocarcinoma (PAAD), rectum adenocarcinoma (READ) and gastric adenocarcinoma (STAD) projects were downloaded from the National Cancer Institute’s Genomic Data Commons Data Portal (GDC), which is part of TCGA [26]. The COAD and READ projects were merged into the CRC project. For the selected probes and panels, the beta values were analyzed for each sample across all projects. These probes and panels were evaluated based on the number of individual samples they successfully identified within the investigated project and the comparison projects, using their beta values as the detection criterion. A beta value of 0.3 was selected as the cut-off criterion for labelling a sample as methylated (which corresponds to 30% methylation in an MS-HRM experiment), which is in line with the cut-off values chosen for MS-HRM. The data for the bioinformatics analysis were collected and processed using the R software environment (version 4.3.0) [27].

### 2.5. Statistical Analysis

The probes selected for each cancer type were combined into a panel. To determine the methylation status of a sample, at least one probe within the panel needed to exceed the methylation threshold of 30%. If the methylation levels of all probes in the panel were below this threshold, the sample was classified as unmethylated.

For the GAC and LUAD panels, sensitivity, specificity, positive predictive value (PPV), negative predictive value (NPV) and diagnostic accuracy were calculated to differentiate between the cancer of interest and paired normal tissue samples (primary cancer), and between all included cancer types and all included cancer types and normal tissues. This statistic was calculated for MS-HRM results, the GEO dataset and the TCGA dataset. Statistics were performed using the R package epiR [28]. To further evaluate the overall diagnostic accuracy of our panels, we performed ROC curve analysis. Experimental MS-HRM methylation data from GAC, LUAD, HCC, CCA, CRC, PDAC and all paired normal tissue samples were included in the ROC curve analysis. The methylation of each sample was converted to a binary positive or negative value based on the predetermined methylation thresholds for each panel and biomarker. We calculated the area under the curve (AUC) together with the 95% confidence interval and the corresponding *p*-value. The DeLong method was applied to calculate the confidence intervals, while the *p*-value was calculated using the Z-score to test whether the AUC was significantly different from 0.5. The analysis was performed with the R package pROC [29,30].

### 2.6. Annotation

Annotation of the HM450 probes was performed using Ensemble Release 110 (Table 1) [31].

## 3. Results

The panels constructed and tested in this study were selected from our previous research [6]. In summary, we conducted a comprehensive bioinformatics analysis of publicly available data from The Cancer Genome Atlas (TCGA), which included 2609 samples of primary tumors from TCGA projects, with diagnoses of breast invasive carcinoma (BRCA), colorectal carcinoma (COADREAD), LUAD, STAD, PAAD, LIHC and CHOL. Additionally, we analyzed 244 samples of normal tissues from the corresponding organs. These samples comprised the discovery set, where we proposed panels of CpG sites to differentiate among the mentioned diagnoses.

**Figure 3:**
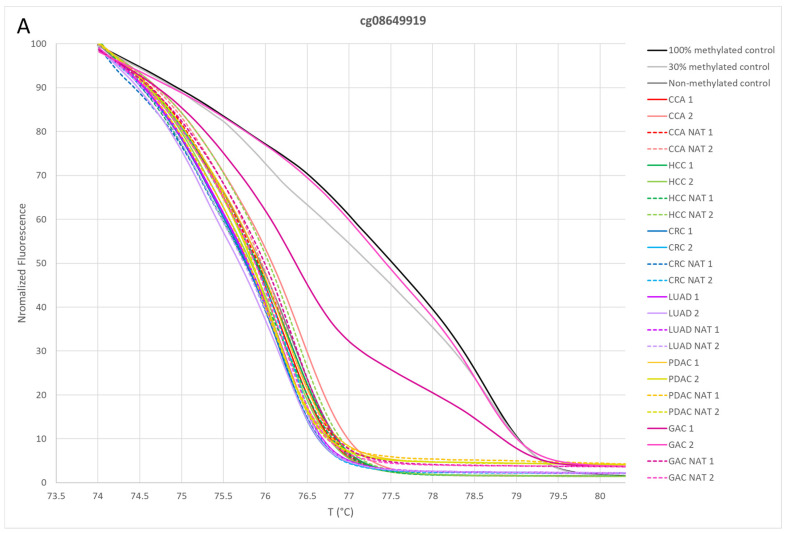

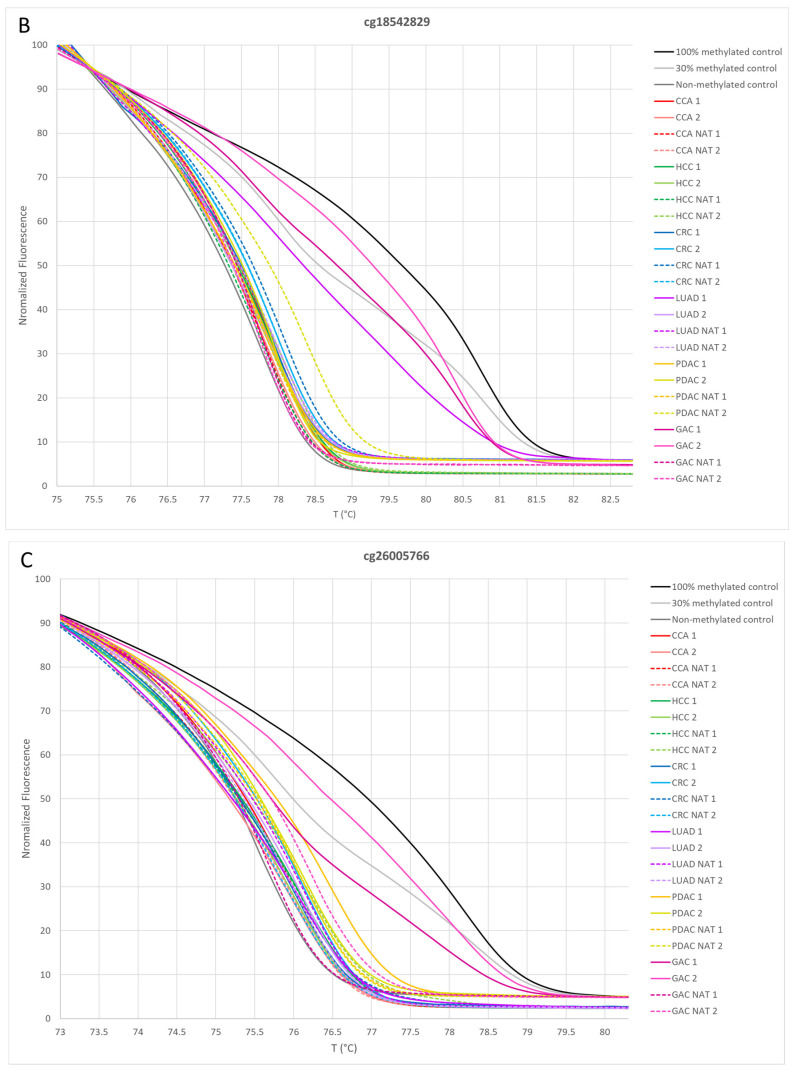
Normalization plots for two paired samples from each cancer type, including cholangiocarcinoma, hepatocellular carcinoma, colorectal carcinoma, lung adenocarcinoma, pancreatic adenocarcinoma and gastric adenocarcinoma, along with their paired normal adjacent tissues and methylation controls, tested on the gastric adenocarcinoma panel. The panel includes the following probes: (**A**) cg08649919, (**B**) cg18542829 and (**C**) cg26005766. CCA, cholangiocarcinoma; HCC, hepatocellular carcinoma; CRC, colorectal carcinoma; LUAD, lung adenocarcinoma; PDAC, pancreatic adenocarcinoma; GAC, gastric adenocarcinoma; NAT, normal adjacent tissue.

The panels for differentiating GAC and LUAD from other common adenocarcinomas were experimentally validated in this study. To test the selected methylation biomarkers included in panels, we experimentally determined the methylation status of 178 samples with MS-HRM. An example of the MS-HRM results and analysis is shown in Figure 2. The evaluation of the methylation status for samples in the GAC panel is presented in Figure 3.

A sample was classified as detected by the panel if at least one probe in the panel was determined to be methylated. For the GAC panel, probe cg08649919 identified GAC 2 as methylated, while GAC 1 was unmethylated (Figure 3A). Probe cg18542829 determined both GAC 1 and GAC 2 as methylated (Figure 3B). Similarly, probe cg26005766 identified GAC 2 as methylated and GAC 1 as unmethylated (Figure 3C). Based on these results, both GAC 1 and GAC 2 samples were classified as positive by the GAC panel, while the remaining samples were classified as negative.

**Figure 4:**
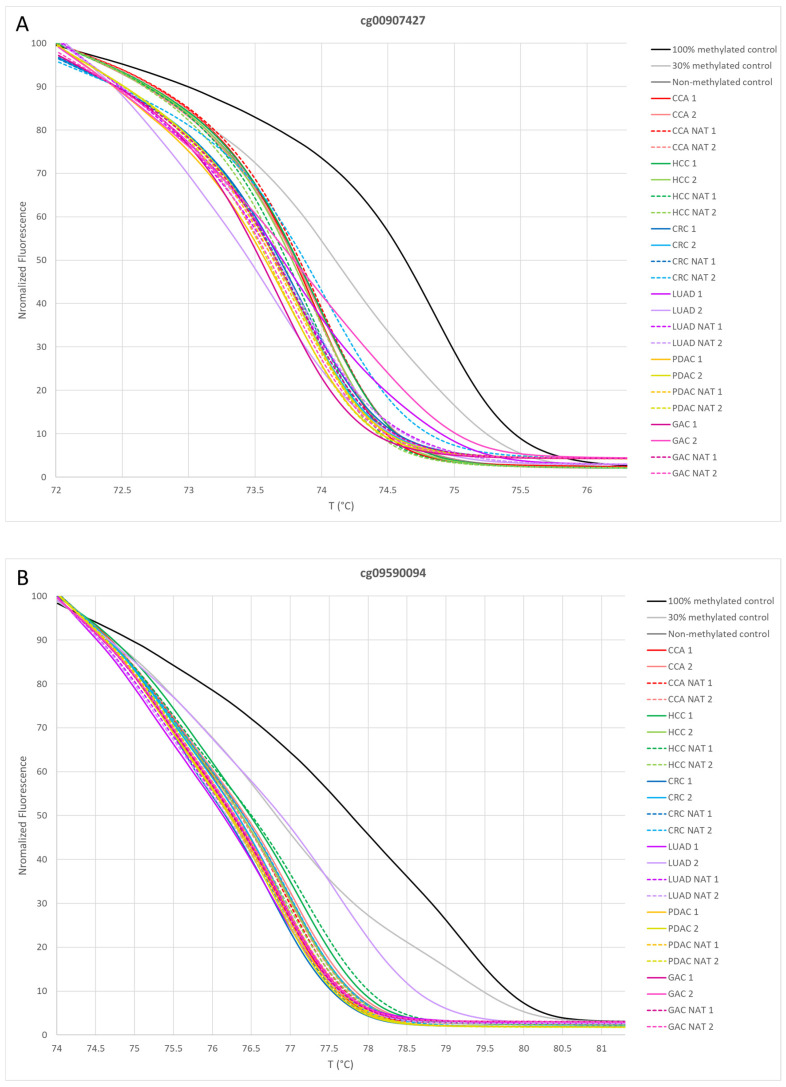

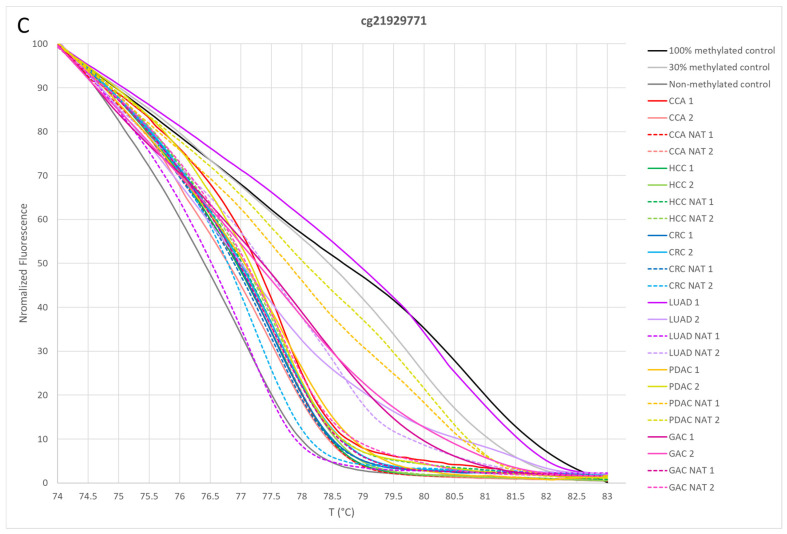
Normalization plots for two paired samples from each cancer type, including cholangiocarcinoma, hepatocellular carcinoma, colorectal carcinoma, lung adenocarcinoma, pancreatic adenocarcinoma and gastric adenocarcinoma, along with their paired normal adjacent tissues and methylation controls, tested on the lung adenocarcinoma panel. The panel includes the following probes: (**A**) cg00907427, (**B**) cg09590094 and (**C**) cg21929771. CCA, cholangiocarcinoma; HCC, hepatocellular carcinoma; CRC, colorectal carcinoma; LUAD, lung adenocarcinoma; PDAC, pancreatic adenocarcinoma; GAC, gastric adenocarcinoma; NAT, normal adjacent tissue.

The evaluation of methylation status for samples in the LUAD panel is presented in Figure 4, using the same approach as for the GAC panel. Probe cg00907427 identified both LUAD 1 and LUAD 2 as unmethylated (Figure 4A). Probe cg09590094 identified LUAD 2 as methylated (Figure 4B), while probe cg21929771 identified LUAD 1 as methylated (Figure 4C). As a result, both LUAD 1 and LUAD 2 samples were classified as positive by the LUAD panel, while the other samples were classified as negative.

To further validate these panels, we used publicly available samples of 395 primary tumor samples and 378 normal tissue samples from the GEO database, and 1833 primary tumor samples and 148 normal tissue samples from TCGA database.

To evaluate the diagnostic potential of the selected probes and panels for GAC and LUAD, we calculated the sensitivity, specificity, accuracy, PPV and NPV for each dataset. The number of methylated samples per diagnosis from the MS-HRM experiment and the GEO and TCGA datasets are shown in Table 2 and Table 3. The comparison of mentioned statistical measures between the datasets is shown in Table 4 and Table 5.

The GAC panel exhibited a sensitivity of 78.6% (11/14), 83.9% (26/31) and 83.0% (328/395) for GAC tumor samples in the MS-HRM experiment, GEO and TCGA datasets, respectively (Table 4). The LUAD panel had a high detection rate for LUAD samples with a sensitivity of 93.3% (14/15) in the MS-HRM experiment, a sensitivity of 61.1% (11/18) in the GEO dataset and a sensitivity of 88.1% (358/454) in the TCGA dataset (Table 5).

The GAC panel comprising the *CABIN1*, *ABCB1* and *TPD52L1* genes reached a sensitivity of 78.6%, a specificity of 92.9% and an accuracy of 85.7% for GAC in the MS-HRM experiment. Moreover, it differentiated between the included tumors with a sensitivity of 78.6%, a specificity of 86.6% and an accuracy of 89.9%, and between all tumors and normal tissues with a specificity of 90.9% and an accuracy of 90.2% (Table 4). Verification with the TCGA and GEO datasets confirmed the ability of the GAC panel to differentiate between GAC and NAT with similar sensitivity (83.9% and 83.0%, respectively). The GEO dataset yielded higher accuracy and specificity for GAC, all tumor samples and all samples. We would also like to point out that the TCGA specificity for GAC with NAT is not representative, as the only available project for the selected probes for NAT consisted of only two samples.

The LUAD panel comprising the *HNRNPR*, *MICAL3* and *PTPRU* genes reported a higher sensitivity in MS-HRM experiment (93.3%) than the bioinformatics results from GEO and TCGA databases (61.1% and 88.1%). In contrast, specificity and diagnostic accuracy for all tumor samples (90.8% and 90.6%) and specificity and diagnostic accuracy for all samples (93.4% and 93.1%) were highest in the TCGA dataset (Table 5). The GEO dataset showed lower sensitivity and accuracy compared to the HRM and TCGA results but similar specificity for all tumor samples and all samples. The specificity for differentiating primary cancer from paired NAT was low in the verification datasets (Table 5).

**Figure 5:**
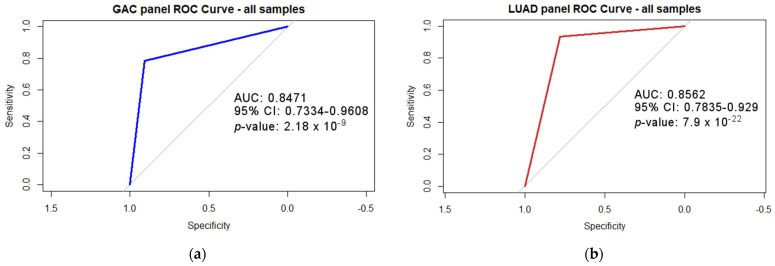
The Receiver operating characteristic (ROC) for GAC and LUAD panels. The area under the curve (AUC), a 95% confidence interval (95% CI) and a *p*-value were calculated. (**a**) GAC panel ROC curve. (**b**) LUAD panel ROC curve.

To evaluate the overall diagnostic accuracy of GAC and LUAD panels in differentiating between all included tumors and NATs, we performed a ROC curve analysis. We calculated the AUC, 95% confidence interval and *p*-values of the ROC curves. The ROC curve analysis included the methylation data from GAC, LUAD, HCC, CCA, CRC and PDAC samples, as well as paired NATs included in the MS-HRM experiment. Our panels yielded an AUC of 0.8471 for the GAC panel and an AUC of 0.8562 for the LUAD panel, and were statistically significant. The results of the ROC curve analyses are presented in Figure 5.

## 4. Discussion

In this study, we experimentally validated diagnostic DNA methylation biomarkers using 178 FFPE tissue samples based on a previously published bioinformatics study employing MS-HRM [6]. We complemented this with a secondary bioinformatics analysis utilizing publicly available DNA methylation data from the TCGA and GEO databases, specifically from the HM450 and EPIC platforms.

With this study, we have effectively translated the bioinformatically identified methylation biomarkers from the HM450 and EPIC platforms to the simpler MS-HRM platform. The experimental MS-HRM results were consistent with the bioinformatics data from the GEO and TCGA databases (Table 2, Table 3, Table 4 and Table 5). Although some results differed between the methods, we confirmed the robustness and comparability of these methods. The reasons for the observed differences may vary. In some cases, the lack of data could lead to biased results (e.g., only two GAC normal tissue samples were available in the TCGA dataset, which are not representative). Moreover, the differences between methods could lead to the observed differences in methylation levels, as MS-HRM covers broader target regions than HM450 and EPIC probes, which may lead to different incorporations concerning the number of CpG sites.

Both panels successfully differentiate GAC and LUAD from other included adenocarcinomas. Good sensitivities, specificities and diagnostic accuracies among the panels were achieved (Table 4 and Table 5). Although the PPV was lower in some groups, the NPV remained high (Table 4 and Table 5). In addition, the ROC curves provided deeper insights into the performance of the diagnostic tests and showed the good discriminatory ability of the GAC and LUAD panels with AUC values between 0.8 and 0.9.

The promoter regions of the three genes were included in each panel. The GAC panel includes the genes *CABIN1*, *ABCB1* and *TPD52L1*, and the LUAD panel includes the genes *HNRNPR*, *MICAL3* and *PTPRU*. The studied genes are involved in cancer development through the regulation of signaling pathways such as p53, ErbB, MAP3K5 and Wnt/β-catenin, the regulation of cell cycle and apoptosis, the regulation of inflammatory response, the regulation of lipid metabolism, the regulation of cytoskeletal dynamics and the regulation of cell signaling [32,33,34,35,36,37,38,39,40,41,42,43,44,45,46]. In addition, overexpression of some genes, including *ABCB1*, *TPD52L1*, *HNRNPR* and *MICAL3*, is associated with poor prognosis in various cancers [35,38,39,40]. Some of the genes are also interesting prospects for their use in therapeutic areas (e.g., *MICAL3* for its role in promoting cancer cell migration and invasion, and *PTPRU* to restore its function as a tumor suppressor), while others are known for their involvement in multidrug resistance in cancer (e.g., *CABIN1* and *ABCB1*) [33,35,36,40,45]. Information about included genes and their main functions in cancer is available in Table S2.

The limitation of our study relates to the small sample size for individual cancer types in the experimental validation. To further evaluate the reliability and efficacy of the proposed panels, they should be tested on a larger sample cohort. The same applies to the validation with bioinformatics data from the GEO and TCGA databases, as only a limited number of samples were available for some sample groups (e.g., only two GAC NAT samples were available in the TCGA database). This could have an impact on the results, as the data may not be representative of the whole sample group, due to the small number of samples. If available, additional bioinformatics data for these groups would allow further confirmation of our experimental results.

To extend our findings, we plan to test our panels on GAC liver metastases and LUAD liver metastases to evaluate the maintenance of methylation in cancer progression. We also believe that the selected biomarkers hold the potential to be tested and detected in cell-free DNA (cfDNA) from GAC and LUAD patients. Ren et al. have already successfully demonstrated that hypermethylation of two of our selected markers, *CABIN1* and *ABCB1*, could be detected in cfDNA from GAC patients and could differentiate GAC from HCC and CRC, as well as from healthy patients [47]. This suggests the biomarkers could be viable in non-invasive diagnostics for various cancers.

In this study, we acknowledge that the tumor samples are derived from known primary sites, allowing for the differentiation between adenocarcinomas based on their organ of origin. However, as diagnostic techniques evolve, the importance of non-invasive approaches, such as liquid biopsies, will become increasingly relevant. In future clinical settings, identifying the source of adenocarcinomas from blood or other body fluids will be crucial for early diagnosis and personalized treatment plans, particularly when the primary tumor site is unknown. The methylation biomarkers validated in this study could serve as a foundation for such efforts, enabling clinicians to detect and localize tumors from cfDNA in blood samples. This approach could revolutionize cancer diagnostics by allowing early detection and guiding further investigation to the specific organ system involved, thereby improving patient outcomes.

## 5. Conclusions

We successfully experimentally validated six bioinformatically identified methylation biomarkers using MS-HRM, combined into GAC-specific and LUAD-specific panels, and tested them on HM450 and EPIC data from GEO and TCGA databases. The GAC-specific and LUAD-specific panels showed good sensitivities, specificities and diagnostic accuracies in the experimental MS-HRM and bioinformatics results, making it possible to differentiate them from other common adenocarcinomas and paired healthy tissues. In conclusion, we successfully translated bioinformatically identified methylation biomarkers from the HM450 and EPIC platforms to the simpler MS-HRM platform, and demonstrated good consistency between them. This study demonstrates the potential of using diagnostic methylation panels to differentiate GAC or LUAD from other common adenocarcinomas and healthy tissues.

## Figures and Tables

**Table 1:** Primer sequences for MS-HRM analysis of the gastric and lung adenocarcinoma panel.

Primer Sequence		Probe	Gene	Annealing T (°C)
Forward	Reverse			
GAC panel				
5′-TTGTAGTGAGAGTTGTATTTATTTTG-3′	5′-ACTTTTACCRACCAATCC-3′	cg08649919	*CABIN1*	58
5′-GTTGTTGTTAAGGAYGTTGGGT-3′	5′-CRCTATAAAACCCCRCAT-3′	cg18542829	*ABCB1*	57
5′-TTYGAGGTTAGGTGAAYGTTA-3′	5′-CRAAAAACAAAAATCRCTCC-3′	cg26005766	*TPD52L1*	57
LUAD panel				
5′-AAATTGTATTTGGTGTATTTGTATAAT-3′	5′-AAAAAATACAAATCTCCTACAAAA-3′	cg00907427	*HNRNPR*	58
5′-GTTAGTAGGTTTTTGGYGAAG-3′	5′-AACCRCTACTCCTAATAAACTCAT-3′	cg09590094	*MICAL3*	58
5′-GTTTTTAATTTYGYGGAGTT-3′	5′-CRCCCCTCRAAAATAAAAT-3′	cg21929771	*PTPRU*	58

GAC, gastric adenocarcinoma; LUAD, lung adenocarcinoma.

**Table 2:** Frequencies of methylated primary tumors and normal adjacent tissues for gastric adenocarcinoma specific probes. A sample was labeled as methylated if it exhibited a methylation level of 30% or higher.

GAC Panel	Primary Cancer	MS-HRM		GEO		TCGA	
		TP (no. M/no. U)	NAT (no. M/no. U)	TP (no. M/no. U)	NAT (no. M/no. U)	TP (no. M/no. U)	NAT (no. M/no. U)
cg26005766 cg18542829 cg08649919	HCC	1/14	0/15	2/112	0/51	10/367	1/49
	CCA	2/13	0/15	0/51	0/16	0/36	0/9
	CRC	5/10	1/14	0/22	0/22	82/305	1/44
	PDAC	1/14	2/13	4/11	0/12	38/146	0/10
	GAC	11/3	1/13	26/5	8/141	328/67	1/1
	LUAD	1/14	1/14	2/16	0/44	38/416	0/32

MS-HRM, Methylation-Sensitive High Resolution Melt analysis; GEO, Gene Expression Omnibus; TCGA, The Cancer Genome Atlas; TP, primary solid tumor; NAT, normal adjacent tissues; no. M, number of methylated samples; no. U, number of unmethylated samples; HCC, hepatocellular carcinoma; CCA, cholangiocarcinoma; CRC, colorectal carcinoma; PDAC, pancreatic adenocarcinoma; GAC, gastric adenocarcinoma; LUAD, lung adenocarcinoma.

**Table 3:** Frequencies of methylated primary tumors and normal adjacent tissues for lung adenocarcinoma specific probes. A sample was labeled as methylated if it exhibited a methylation level of 30% or higher.

LUAD Panel	Primary Cancer	MS-HRM		GEO		TCGA	
		TP (no. M/no. U)	NAT (no. M/no. U)	TP (no. M/no. U)	NAT (no. M/no. U)	TP (no. M/no. U)	NAT (no. M/no. U)
cg26005766 cg18542829 cg08649919	HCC	3/12	2/13	4/110	0/51	28/349	0/50
	CCA	4/11	5/10	13/38	0/16	6/30	0/9
	CRC	4/11	2/13	9/104	0/103	35/352	1/44
	PDAC	6/9	3/12	3/29	1/14	15/169	0/10
	GAC	5/9	0/14	5/26	0/149	87/308	0/2
	LUAD	14/1	2/13	11/7	39/5	358/96	32/0

MS-HRM, Methylation-Sensitive High Resolution Melt analysis; GEO, Gene Expression Omnibus; TCGA, The Cancer Genome Atlas; TP, primary solid tumor; NAT, normal adjacent tissues; no. M, number of methylated samples; no. U, number of unmethylated samples; HCC, hepatocellular carcinoma; CCA, cholangiocarcinoma; CRC, colorectal carcinoma; PDAC, pancreatic adenocarcinoma; GAC, gastric adenocarcinoma; LUAD, lung adenocarcinoma.

**Table 4:** Statistical analysis for the gastric adenocarcinoma-specific panel for the MS-HRM experiment, the GEO dataset and the TCGA dataset. Sensitivity, specificity, positive predictive value, negative predictive value and diagnostic accuracy were calculated to differentiate between GAC and paired normal tissue samples (GAC with NAT), GAC from all included cancer types (All tumor samples) and GG from included cancer types and normal tissues (All samples).

GAC Panel	MS-HRM			GEO			TCGA		
	GAC with NAT ^+^	All Tumor Samples ^++^	All Samples ^+++^	GAC with NAT ^+^	All Tumor Samples ^++^	All Samples ^+++^	GAC with NAT ^+^	All Tumor Samples ^++^	All Samples ^+++^
Sensitivity	78.6%	78.6%	78.6%	83.9%	83.9%	83.9%	83.0%	83.0%	83.0%
Specificity	92.9%	86.6%	90.9%	94.6%	96.4%	96.9%	50.0%	88.3%	89.2%
Accuracy	85.7%	89.9%	90.2%	92.8%	94.8%	96.1%	82.9%	87.2%	88.0%
PPV	91.7%	52.4%	42.3%	76.5%	76.5%	61.9%	99.7%	66.1%	65.7%
NPV	81.3%	98.0%	98.1%	96.6%	97.7%	99.0%	1.5%	95.0%	95.5%

^+^, GAC with NAT refers to gastric adenocarcinoma and its NATs; ^++^, All tumor samples include GAC, LUAD, HCC, CCA, PDAC and CRC tumor samples; ^+++^, All samples include GAC, LUAD, HCC, CCA, PDAC and CRC tumor samples along with their paired NATs. GAC, gastric adenocarcinoma; MS-HRM, Methylation-Sensitive High Resolution Melt analysis; GEO, Gene Expression Omnibus; TCGA, The Cancer Genome Atlas; NAT, normal adjacent tissues; PPV, positive predictive value; NPV, negative predictive value.

**Table 5:** Statistical analysis for the LUAD panel for the MS-HRM experiment, GEO dataset and TCGA dataset. Sensitivity, specificity, positive predictive value, negative predictive value and diagnostic accuracy were calculated to differentiate between LUAD and paired normal tissue samples (LUAD with NAT), between LUAD from all included cancer types (All tumor samples), and LUAD from all included cancer types and normal tissues (All samples).

LUAD Panel	MS-HRM			GEO			TCGA		
	LUAD with NAT ^+^	All Tumor Samples ^++^	All Samples ^+++^	LUAD with NAT ^+^	All Tumor Samples ^++^	All Samples ^+++^	LUAD with NAT ^+^	All Tumor Samples ^++^	All Samples ^+++^
Sensitivity	93.3%	93.3%	93.3%	61.1%	61.1%	61.1%	88.1%	88.1%	88.1%
Specificity	86.7%	70.3%	77.9%	11.4%	90.0%	89.7%	35.0%	90.8%	93.4%
Accuracy	90.0%	74.2%	79.2%	25.8%	88.6%	89.0%	71.0%	90.6%	93.1%
PPV	87.5%	38.9%	28.0%	22.0%	24.4%	12.9%	74.0%	52.1%	43.5%
NPV	92.9%	98.1%	99.2%	41.7%	97.8%	98.9%	58.3%	98.5%	99.3%

^+^, LUAD with NAT refers to gastric adenocarcinoma and its NATs; ^++^, All tumor samples include LUAD, GAC, HCC, CCA, PDAC and CRC tumor samples; ^+++^, All samples include LUAD, GAC, HCC, CCA, PDAC and CRC tumor samples along with their paired NATs. LUAD, lung adenocarcinoma; MS-HRM, Methylation-Sensitive High Resolution Melt analysis; GEO, Gene Expression Omnibus; TCGA, The Cancer Genome Atlas; NAT, normal adjacent tissues; PPV, positive predictive value; NPV, negative predictive value.

## Data Availability

The data presented in this study are available in this article and Supplementary Materials.

## Supplementary Materials

The following supporting information can be downloaded at: https://www.mdpi.com/article/10.3390/cancers16234000/s1, Table S1. The demographic data of all included patients including gender, age, diagnosis, histologic subtype, histologic grade and TNM classification of malignant tumors; Table S2. An overview of the genes included.
