# Supplementary material for: DNA Methylation Panels for the Differentiation of Lung and Gastric Adenocarcinomas from Other Common Primary Adenocarcinomas
Source: Cancers (Basel). 2024 Nov 29;16(23):4000. doi: 10.3390/cancers16234000 (PMC11640184; doi:10.3390/cancers16234000)
Supplement: Supplementary file 1 [file cancers-16-04000-s001.zip › cancers-3293406-supplementary.pdf]

Table S1. The demographic data of all included patients including gender, age, diagnosis, histologic subtype, histologic grade and TNM classification of malignant tumors. TNM staging was performed in accordance with the UICC 8th Edition 2017.

Patient ID	Gender	Age	Diagnosis	Histologic subtype	Histologic grade	TNM Classification
1	F	72	LUAD	Mixed predominantly solid adenocarcinoma	Poorly differentiated	pT1c N0 Mx
2	M	73	LUAD	Papillary adenocarcinoma	Poorly differentiated	pT2b N0 Mx
3	F	67	LUAD	Papillary adenocarcinoma	Moderately differentiated	pT1b N2 Mx
4	F	74	LUAD	Mixed predominantly papillary adenocarcinoma	Poorly differentiated	pT2a N1 Mx
5	F	62	LUAD	Papillary adenocarcinoma	Moderately differentiated	pT2a N1 Mx
6	F	72	LUAD	Papillary adenocarcinoma	Moderately differentiated	pT1c N0 Mx
7	F	60	LUAD	Mixed predominantly papillary adenocarcinoma	Poorly differentiated	pT3 N0 M0
8	M	59	LUAD	Solid adenocarcinoma	Poorly differentiated	pT2 N1 Mx
9	F	58	LUAD	Papillary adenocarcinoma	Moderately differentiated	pT2a N0 Mx
10	M	64	LUAD	Mixed predominantly papillary adenocarcinoma	Poorly differentiated	pT2a N0 Mx
11	F	71	LUAD	Papillary adenocarcinoma	Poorly differentiated	pT2a N0 Mx
12	M	54	LUAD	Solid adenocarcinoma	Poorly differentiated	pT4 N1 Mx
13	M	72	LUAD	Mixed predominantly micropapillary adenocarcinoma	Poorly differentiated	pT2a N0 Mx
14	M	67	LUAD	Papillary adenocarcinoma	Moderately differentiated	pT1c Nx Mx
15	M	48	LUAD	Papillary adenocarcinoma	Moderately differentiated	pT2a N0 Mx
16	F	75	GAC	Mucinous adenocarcinoma (WHO)<br>Intestinal adenocarcinoma (Lauren)	Poorly differentiated	pT4 N1 M1
17	F	71	GAC	Poorly cohesive adenocarcinoma (WHO)<br>Diffuse adenocarcinom (Lauren)	Poorly differentiated	pT3 N2 M1
18	M	71	GAC	Mixed poorly cohesive and tubular adenocarcinoma (WHO)<br>Mixed adenocarcinoma (Lauren)	Poorly differentiated	pT4a N3b Mx
19	F	50	GAC	Mixed poorly cohesive and tubular adenocarcinoma (WHO)<br>Intestinal and diffuse adenocarcinoma (Lauren)	Poorly differentiated	pT2 N3a Mx
20	F	78	GAC	Intestinal and poorly cohesive adenocarcinoma (WHO)<br>Mixed adenocarcinoma (Lauren)	Poorly differentiated	pT4a N3a Mx
21	F	63	GAC	Poorly cohesive, partially signet ring cell adenocarcinoma (WHO)<br>Diffuse adenocarcinoma (Lauren)	Poorly differentiated	pT4a N3b M1
22	M	57	GAC	Mixed tubular and poorly cohesive adenocarcinoma (WHO)<br>Intestinal and diffuse adenocarcinoma (Lauren)	Poorly differentiated	pT1a N0 Mx
23	F	68	GAC	Mixed tubular and poorly cohesive adenocarcinoma (WHO)<br>Mixed adenocarcinoma (Lauren)	Poorly differentiated	pT4a N2 Mx

24	F	32	GAC	Mixed tubular and poorly cohesive adenocarcinoma (WHO)<br>Diffuse adenocarcinoma (Lauren)	Poorly differentiated	pT4a N3a Mx
25	M	64	GAC	Mixed tubular and papillary adenocarcinoma (WHO)<br>Mixed predominantly intestinal adenocarcinoma (Lauren)	Moderately differentiated	pT3 N1 Mx
26	M	50	GAC	Poorly cohesive signet ring cell adenocarcinoma (WHO)<br>Diffuse adenocarcinoma (Lauren)	Poorly differentiated	pT3 N0 Mx
27	M	67	GAC	Mixed tubular and poorly cohesive adenocarcinoma (WHO)<br>Mixed predominantly intestinal adenocarcinoma (Lauren)	Poorly differentiated	pT1b N0
28	M	84	GAC	Tubular adenocarcinoma (WHO)<br>Intestinal adenocarcinoma (Lauren)	Poorly differentiated	pT4b N2 Mx
29	M	77	GAC	Mixed tubular and poorly cohesive adenocarcinoma (WHO)<br>Intestinal and diffuse adenocarcinoma (Lauren)	Poorly differentiated	pT4a N3a M1
30	M	61	HCC	Macrotrabecular and pseudoglandular carcinoma	Well differentiated	pT1a Nx Mx
31	M	77	HCC	Pseudoglandular carcinoma	Well differentiated	pT1b Nx Mx
32	M	70	HCC	Macrotrabecular carcinoma	Poorly differentiated	pT3 N0 Mx
33	M	70	HCC	Macrotrabecular and pseudoglandular carcinoma	Well differentiated	pT2 N0 Mx
34	M	54	HCC	Macrotrabecular carcinoma	Moderately differentiated	pT1a Nx Mx
35	M	75	HCC	Steatotic carcinoma	Moderately differentiated	pT1b Nx Mx
36	M	70	HCC	Macrotrabecular and pseudoglandular carcinoma	Moderately differentiated	pT2 Nx Mx
37	M	69	HCC	Macrotrabecular carcinoma	Poorly differentiated	pT2 Nx Mx
38	F	77	HCC	Trabecular and steatotic, partially solid carcinoma	Well differentiated	pT2 Nx Mx
39	M	76	HCC	Macrotrabecular and pseudoglandular carcinoma	Poorly differentiated	pT1b Nx Mx
40	F	60	HCC	Macrotrabecular carcinoma with hyaline globules	Poorly differentiated	pT2 Nx Mx
41	M	63	HCC	Pseudoglandular carcinoma	Moderately differentiated	pT1b N0 Mx
42	M	63	HCC	Macrotrabecular carcinoma with hyaline globules	Poorly differentiated	pT2 Nx Mx
43	F	64	HCC	Macrotrabecular carcinoma	Poorly differentiated	pT1 Nx Mx
44	M	60	HCC	Pseudoglandular carcinoma	Well differentiated	pT1a Nx Mx
45	F	81	CCA	Small duct adenocarcinoma	Well differentiated	pT1b Nx Mx
46	F	68	CCA	Small duct adenocarcinoma	Moderately differentiated	pT1a Nx Mx
47	F	64	CCA	Small duct adenocarcinoma	Moderately differentiated	pT2 Nx Mx
48	M	63	CCA	Pancreatobiliary adenocarcinoma	Moderately differentiated	pT2b N0 Mx
49	M	65	CCA	Perihilar adenocarcinoma	Moderately differentiated	pT2a N0 Mx
50	M	60	CCA	Small duct adenocarcinoma	Moderately differentiated	pT1a N0 Mx
51	F	63	CCA	Small duct adenocarcinoma	Moderately differentiated	pT2 N1 Mx
52	F	59	CCA	Small duct adenocarcinoma	Poorly differentiated	pT2 N1 Mx
53	M	55	CCA	Small duct adenocarcinoma	Moderately differentiated	pT2 N1 Mx
54	M	45	CCA	Small duct adenocarcinoma	Moderately differentiated	pT2 N2 Mx

55	M	72	CCA	Small duct adenocarcinoma	Poorly differentiated	pT2 N1 Mx
56	M	69	CCA	Small duct adenocarcinoma	Poorly differentiated	pT2b Nx Mx
57	F	73	CCA	Large duct adenocarcinoma	Moderately differentiated	pT2 N1 Mx
58	F	70	CCA	Small duct adenocarcinoma	Poorly differentiated	pT2b N2 Mx
59	M	69	CCA	Small duct adenocarcinoma	Poorly differentiated	pT2 Nx Mx
60	M	83	CRC	Adenocarcinoma NOS	Poorly differentiated	pT3 N2a Mx
61	F	64	CRC	Adenocarcinoma NOS	Moderately differentiated	pT4b N0 Mx
62	M	82	CRC	Mucinous adenocarcinoma	NA	pT3 N0 Mx
63	F	59	CRC	Adenocarcinoma NOS	Moderately differentiated	pT4a N2b Mx
64	F	72	CRC	Medullary adenocarcinoma	Poorly differentiated	pT4b N1 Mx
65	M	74	CRC	Adenocarcinoma NOS	Moderately differentiated	pT2 N0 M1b
66	F	63	CRC	Adenocarcinoma NOS	Moderately differentiated	pT3 N2b M1a
67	M	66	CRC	Adenocarcinoma NOS	Poorly differentiated	pT4a N2b M1a
68	F	81	CRC	Adenocarcinoma NOS	Poorly differentiated	pT3 N2a M1
69	M	68	CRC	Adenocarcinoma NOS	Poorly differentiated	pT4 N2 M1
70	M	72	CRC	Adenocarcinoma NOS	Moderately differentiated	pT3 N2b M1a
71	F	80	CRC	Adenocarcinoma NOS	Moderately differentiated	pT4a N1b M1a
72	F	75	CRC	Adenocarcinoma NOS	Moderately differentiated	pTx Nx M1
73	M	81	CRC	Adenocarcinoma NOS	Moderately differentiated	pT4a N1b M1a
74	F	44	CRC	Adenocarcinoma NOS	Moderately differentiated	pT4a N2b M1
75	F	58	PDAC	Ductal adenocarcinoma	Poorly differentiated	pT2 N2 Mx
76	M	51	PDAC	Ductal adenocarcinoma	Poorly differentiated	pT2 N1 Mx
77	F	77	PDAC	Ductal adenocarcinoma	Poorly differentiated	pT2 N2 Mx
78	F	76	PDAC	Ductal adenocarcinoma	Moderately differentiated	pT3 N1 Mx
79	F	85	PDAC	Ductal adenocarcinoma	Moderately differentiated	pT2 N0 Mx
80	F	77	PDAC	Ductal adenocarcinoma	Moderately differentiated	pT2 N0 Mx
81	M	69	PDAC	Ductal adenocarcinoma	Poorly differentiated	pT2 N2 Mx
82	M	76	PDAC	Ductal adenocarcinoma	Poorly differentiated	pT2 N1 Mx
83	M	58	PDAC	Ductal adenocarcinoma	Poorly differentiated	pT2 N2 Mx
84	M	70	PDAC	Ductal adenocarcinoma	Poorly differentiated	pT3 N2 Mx
85	F	61	PDAC	Ductal adenocarcinoma	Poorly differentiated	pT3 N1 Mx
86	M	70	PDAC	Ductal adenocarcinoma	Poorly differentiated	pT3 N1 Mx
87	F	80	PDAC	Ductal adenocarcinoma	Poorly differentiated	pT3 N1 Mx
88	F	57	PDAC	Ductal adenocarcinoma	Poorly differentiated	pT3 N1 Mx
89	M	85	PDAC	Ductal adenocarcinoma	Moderately differentiated	pT3 N1 Mx

Legend: M, male; F, female; LUAD, lung adenocarcinoma; GAC, gastric adenocarcinoma; HCC, hepatocellular carcinoma; CCA, cholangiocarcinoma; CRC, colorectal adenocarcinoma; PDAC, pancreatic ductal adenocarcinoma; NOS, not otherwise specified; NA, not applicable.

Table S2. An overview of the genes included. Listed are the gene abbreviation, the full name, the description of the gene functions in healthy tissue, their involvement in cancer and their potential role as oncogenes or tumor suppressor genes.

Gene	Full name	Protein class	Function in healthy tissues	Role in cancer	Oncogene/tumor suppressor	References
CABIN1	Calcineurin-Binding Protein 1	Polyubiquitin-binding protein	Regulatory role in immune responses and gene expression via regulation of calcineurin signaling, role in apoptosis and cell survival, transcriptional regulation and chromatin assembly.	- Maintaining chromatin dynamics and influencing gene expression during cell proliferation, thereby influencing oncogenesis.<br>- Influencing the transcription of genes involved in the cell cycle as a negative regulator of p53/TP53, which may contribute to the dysregulation of proliferation in cancer cells.<br>- Regulation of apoptosis.<br>- Interacts with the ErbB signaling pathway, which may lead to sustained signaling cascades that promote cancer cell survival and proliferation.<br>- Affects the tumor microenvironment.	Tumor suppressor	[32-34, 46]
ABCB1(also known as *MDRP*)	P-glycoprotein 1 (also Multi-drug resistance protein 1)	P-glycoprotein	ATP-binding cassette transporter that actively transports substrates, such as toxins and drugs, across cellular membranes.	- Involved in multidrug resistance in cancer.<br>- Regulated by the Wnt/β-catenin signaling pathway, which increases ABCB1 transcription and activity, thereby promoting drug resistance.	Oncogene	[35-36, 46]
TPD52L1	Tumor Protein D52 Like 1	Intracellular Protein	Involved in proliferation, regulation of the cell cycle, apoptosis, calcium signaling and interaction with other proteins.	- Promotion of cell proliferation.<br>- Possible regulation of apoptosis via an interaction with MAP3K5, which may influence the survival of cancer cells.<br>- Regulation of lipid metabolism, which is an important aspect of cancer metabolism.	Oncogene	[37-38, 46]
HNRNPR	Heterogeneous Nuclear Ribonucleoprotein R	RNA-binding protein	A member of the spliceosome C complex that plays a role in the processing and transport of pre-mRNA.	- Promotes proliferation and metastasis via the CCNB1/CENPF axis and influences key processes such as cell cycle progression and cellular migration.<br>- Is involved in the regulation of alternative splicing, which can influence tumor progression by modulating the expression of various oncogenes and tumor suppressors.	Oncogene	[39, 46]
MICAL3	Microtubule Associated Monooxygenase, Calponin And LIM Domain Containing 3	Monooxygenase	Enables actin binding activity. Participates in actin filament depolymerization, regulation of exocytosis and cellular signaling.	- Regulation of cytoskeletal dynamics affecting cell shape and motility, which is crucial for processes like invasion and metastasis in cancer cells.<br>- May amplify the signals of growth factor receptors, which are involved in cell proliferation and survival. Its function can lead to enhanced oncogenic signaling.<br>- Its expression has been associated with markers of EMT.	Oncogene	[40-42, 46]
PTPRU	Protein Tyrosine Phosphatase Receptor Type U	Receptor-type protein tyrosine phosphatase	By dephosphorylation of tyrosine residues on proteins involved in cellular signaling and maintenance of normal physiological processes, including growth, differentiation and apoptosis.	- Regulation of cell signaling such as dephosphorylation of specific proteins involved in cell signaling pathways, thereby influencing cellular processes such as proliferation, differentiation and migration.<br>- Downregulation or loss often correlates with tumor progression in various cancers, suggesting that PTPRU prevents uncontrolled cell growth and maintains normal cellular function.	Tumor suppressor	[43-46]
